# Dry mouth in patients with a life-limiting condition or frailty: a study protocol for two intervention studies and a nested qualitative sub-study (the Dry mOuth Project, DROP)

**DOI:** 10.1186/s12904-023-01242-0

**Published:** 2023-08-23

**Authors:** Annelot I. van der Meulen, Evelien P. J. G. Neis, Ellen J. M. de Nijs, Bénédicte J. E. G. Coenegracht, Arianne Stoppelenburg, Marieke H. J. van den Beuken-van Everdingen, Yvette M. van der Linden

**Affiliations:** 1 Center of Expertise in Palliative Care, Leiden University Medical Center, Leiden, Netherlands; 2 Center of Expertise in Palliative Care, Maastricht University Medical Center, Maastricht, Netherlands; 3 Integrative Supportive and Palliative Care Team, Zuyderland Medical Center, Heerlen, Netherlands; 4 Netherlands Comprehensive Cancer Organization (IKNL), Utrecht, Netherlands

**Keywords:** Xerostomia, Dry Mouth, Palliative Care, End-of-Life-Care, Randomized Controlled Trial, Pilocarpine, Placebo, Education

## Abstract

**Background:**

Despite its prevalent and impactful nature, dry mouth remains an underexposed and undertreated symptom in patients with a life-limiting condition or frailty. The main contributing factors are a lack of awareness and knowledge amongst both healthcare professionals and patients, and a scarcity of effective, evidence-based interventions. In the DRy mOuth Project (DROP), we address these factors by investigating both a non-pharmacological and a pharmacological intervention: a nurse-led patient education program and locally applied pilocarpine.

**Methods:**

This intervention-based research project consists of two parallel studies. The non-pharmacological study is a cluster non-randomized controlled trial in 228 palliative nursing home and hospital patients, investigating the effect of structured use of guidelines and of patient education on dry mouth symptoms. This intervention, a nurse-led patient education program (the Mouth Education Program, MEP), will be compared to care as usual, the control. The pharmacological study is a double-blind placebo-controlled randomized trial that examines the effect of locally applied pilocarpine drops in 120 patients with dry mouth symptoms. Both studies use the same mixed-methods study design, in which the primary outcome is the clinical response to the intervention at 4 weeks, as measured by a dry mouth severity score (numeric rating scale from 0 to 10). Other outcomes, as measured by questionnaires over a 12-week follow-up period, include durability of the effect, impact on quality of life and, adherence and acceptability of the intervention. In addition, the feasibility and cost-effectiveness are evaluated by means of questionnaires and focus groups with healthcare professionals, and interviews with patients.

**Discussion:**

This study investigates the effectiveness and feasibility of two interventions for dry mouth symptoms in patients with life-limiting conditions or frailty. Due to the large-scale and mixed-method nature of the study, this study will also improve our understanding of dry mouth and its relating factors and of the patients’ and healthcare professionals’ experiences with symptoms, care and guidelines of dry mouth, including any perceived barriers and facilitators.

**Trial registration:**

NCT05964959 & NCT05506137.

## Background

Xerostomia or dry mouth is a severely impactful yet underacknowledged symptom in patients with a life-limiting illness or frailty. It is often associated with alterations in the quality and quantity of saliva, which leads to functional alterations like halitosis, burning sensations, altered taste perception, pain, and difficulty in eating, swallowing and speaking [1, 2]. As such, xerostomia causes physical and emotional discomfort, thereby significantly reducing quality of life [2, 3].

Dry mouth complaints are very common in both older patients and patients in the last phase of their lives [4]. Xerostomia is present in 20 to 60% of the elderly [2, 5, 6], in 50 to 83% of cancer patients receiving palliative care [7–9] and in 60% to 85% of patients with advanced disease [1, 10]. Prevalence of xerostomia highly depends on patient characteristics: important causes are polypharmacy and anticholinergic drugs, aging salivary glands, various chronic diseases, chemotherapy, previous history of radiotherapy to the salivary glands in head and neck cancer and general poor health [2].

Despite its prevalent and impactful nature, dry mouth remains an undertreated symptom in patients due to two main barriers: limited attention to and recognition of dry mouth complaints amongst both health care professionals and patients themselves, and a lack of evidence-based, effective pharmacological interventions[4].

As for the first barrier, health care professionals are often aware of the importance of oral care and high prevalence of dry mouth symptoms in the palliative phase but experience difficulty in addressing and treating dry mouth in clinical practice [4, 11]. An important factor is a lack of training, resulting in limited knowledge and discomfort with performing oral care and oral health consultations [12–14]. Other contributing factors are a lack of uniform guidelines and protocols, and an unclear division of responsibilities [12, 13]. Furthermore, patients are often not aware of the options to ease their symptoms and do not know which healthcare professional they can turn to for their dry mouth problems [4, 14]. Current research, however, shows that participation in education programs about oral care by both patients and professional caregivers can be an effective intervention to improving oral health in general and dry mouth specifically. One meta-analysis showed that education programs for older patients with dry mouth increases oral salivary secretion and improves oral health related quality of life [15], while another meta-analysis showed that education programs for long-term nursing home caregivers increases normal, non-xerostomic oral mucosa and decreases detectable stomatitis in their patients [16].

The second barrier is the absence of effective, pharmacological treatments with limited to no side-effects. Aside from symptom management through lifestyle, dietary and oral hygiene improvement and/or short-lasting saliva substitutes, there is just one (off-label) pharmacological option available in the Netherlands and most other countries: pilocarpine [17–19]. Pilocarpine is a parasympathomimetic and systemic drug that activates muscarinic receptors, thus increasing the secretion of natural saliva and alleviating symptoms of dry mouth. Systemic pilocarpine, however, has been associated with severe side effects, such as excessive transpiration, headaches, dizziness and frequent urination. The severity of these side effects often do not outweigh the benefits of the drug [20]. Therefore, current research focuses on topical administration of pilocarpine in the oral cavity (e.g. lozenges, drops or mouthwash). This has indeed been identified as a potentially effective treatment option for dry mouth with significantly fewer to no adverse side effects [20–22]. In fact, a previous pilot study of our research group has found that locally administered pilocarpine drops are effective in reducing xerostomia complaints with minimal side effects in older patients with xerostomia [21]. While this and other recent studies show promising results for topical administration of pilocarpine in patients with dry mouth, there is currently insufficient high level evidence due to the use of small sample sizes and the lack of double-blind placebo randomized controlled trials.

The DRy mOuth Project (DROP) attempts to contribute to better care for patients with dry mouth in the last phase of life by addressing the above-mentioned barriers with two separate intervention studies. The first barrier, the lack of knowledge and awareness, will be addressed by investigating a nurse-led patient education program in a cluster mixed-methods trial. The second barrier, the lack of an effective pharmacological treatment, will be addressed by investigating a topical form pilocarpine in a double-blind placebo randomized controlled trial.

In this article, we describe the aim, design and procedures for the two studies of the DROP. In both studies, the interventions’ clinical effectiveness, practical feasibility and cost-effectiveness are assessed. The DROP aims to:

1. investigate the effect of both a nurse-led patient education program and a local form of pilocarpine on dry mouth severity and oral health related quality of life;
2. evaluate the feasibility and acceptability of both interventions; and
3. explore current experiences with dry mouth care and dry mouth care guidelines of both patients and health care professionals in palliative care.

## Methods

### Study design

The DROP consists of a non-pharmacological and a pharmacological intervention study.

**Fig. 1:**
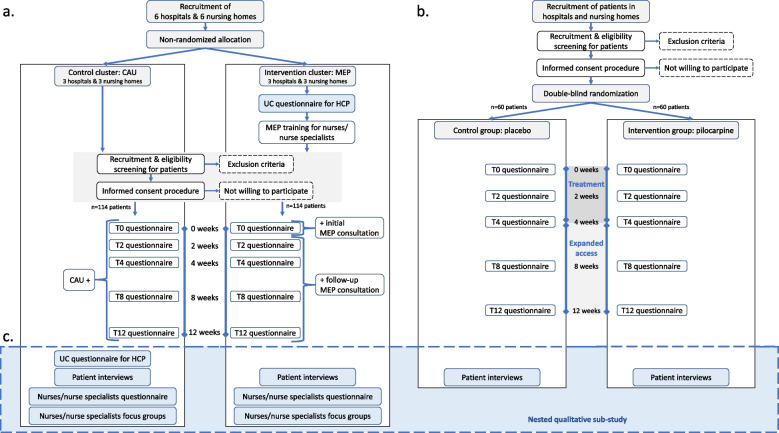
Study procedures for the non-pharmacological (**a**) and pharmacological study (**b**) within the Dry mOuth Project. **a** The Mouth Education Program (MEP) study is a cluster non-randomized trial with a study period of 12 weeks and five visits per patient. **b** The pilocarpine study is a double-blind placebo randomized controlled trial with an active treatment period of four weeks and an optional expanded access period of eight weeks per patient. **c** A nested qualitative sub-study will be conducted during and after both studies. Abbreviations: CAU = care as usual, MEP = Mouth Education Program, UC = usual care; HCP = health care professionals; T0-12 = patient timeline at *t* = 0, *t* = 2, *t* = 4, *t* = 8 and *t* = 12 weeks

Both studies are multi-center, prospective, intervention trials with a nested qualitative sub-study, investigating the effect of the interventions on dry mouth symptoms in patients with a life-limiting illness or frailty (see also Fig. 1).

#### Mouth Education Program (MEP) study

The non-pharmacological study is a cluster non-randomized trial with 228 patients divided over twelve clusters (twelve organisations) in two care settings: a hospital setting with six clusters and a nursing home setting with six clusters. Within each care setting, three clusters will be allocated to the intervention arm on a first come, first serve basis (114 patients), after which three clusters will be allocated to the control arm (114 patients). The intervention, the Mouth Education Program (MEP), will be compared to the control, care as usual (CAU) over a twelve-week period. A cluster non-randomized design was chosen to reduce between-condition contamination and to improve recruitment and logistic processes [23].

#### Pilocarpine study

The pharmacological study is a four-week double-blind placebo-controlled randomized trial with an eight week unblinded extension treatment period with off-label pilocarpine eye drops with 120 patients from either hospital or nursing home setting. The study intends to evaluate the effect of locally administered oral pilocarpine drops (3 × 5 mg of pilocarpine per day) in reducing complaints of dry mouth in a palliative population at the expense of limited adverse events, as compared to placebo. A double-blind placebo-controlled randomized controlled design was chosen to minimize response bias and achieve a high level of evidence.

### Study population

#### Patients with dry mouth

In both studies, patients in the palliative phase of their disease with moderate to severe dry mouth symptoms will be invited to participate in the study. Eligibility is assessed using the following inclusion criteria: dry mouth severity ≥ 5 on a scale from 0 to 10 (NRS), a life-limiting condition or frailty [24], an age of 18 years or older and possible palliative care needs as measured by the surprise question [25]. In order to fulfill the surprise question, that is “Would you be surprised if the patient died within 12 months?”, the answer must be “No”. Patients with severe cognitive decline, with previous radiotherapy to the head-neck area and/or with Sjögren’s syndrome will be excluded. Patients with a life expectancy of ≤ 4 weeks will also be excluded due to the primary endpoint being set at 4 weeks. Lastly, in the pilocarpine study patients with mobility impairments impeding correct administration of the drops will be excluded.

#### Healthcare professionals (MEP study)

A selection of healthcare professionals working at the hospitals and nursing homes within the MEP study will be asked to answer a questionnaire about current practice in dry mouth care, either before the start of the study (intervention group) or after the start of the study (control group). This group of healthcare professionals consists of nursing home doctors, nursing home nurses and nurse specialists from the hospital-based palliative care consultation teams. In addition, upon completion of the full study period the hospital-based nurse specialists and nursing home nurses leading the patient education consultations in the intervention group will be asked to participate in a questionnaire and in focus groups regarding the feasibility of the MEP. Similarly, nurses and nurse specialists from the control group will be asked to participate in a focus group to further evaluate their experiences with current care practices and guidelines.

### Intervention and control

#### MEP study

The MEP is a patient education program and has been developed by our team to increase the use of guidelines by nurses in both hospitals and nursing homes, to strengthen awareness and knowledge in both nurses and patients and to improve management of dry mouth symptoms.

The structure and methods of the MEP were built on previous experience with patient education programs [26]. The content of the patient education program is based on existing palliative care guidelines [17–19] and has been reviewed by our multidisciplinary expert group, including palliative care researchers, an elderly care physician, a radiation oncologist, palliative care nurse specialists, an oral hygienist, a dentist and a patient representative. The program has also been piloted by two independent palliative care nurse specialists to ensure practical feasibility.

**Fig. 2:**
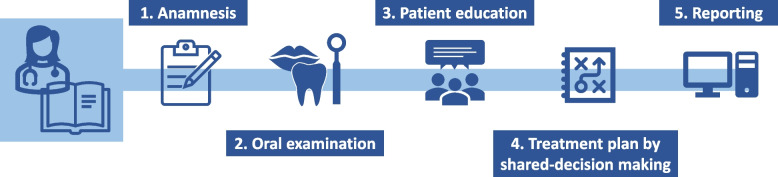
Structure of the standardized MEP consultation. Using the MEP handbook, the trained MEP nurses and nurse specialists will perform a standardized consultation, including: evaluating the dry mouth complaint by anamnesis and oral examination, providing information on dry mouth care (patient education), creating a treatment plan by shared-decision making and reporting all findings and decisions in the patient’s medical records

Table 1 shows the components and contents of the patient education program on dry mouth. The patient education program consists of a training for participating nurses, structured consultations with patients using the MEP handbook and patient information brochures. The MEP handbook is a guidebook, containing step-by-step plans for the initial and follow-up consultations, background information on dry mouth and oral care, conversation pointers and examples of different patient scenarios. To ensure the quality and uniformity of the patient education program, all participating nurses from the nursing homes and nurse specialists from the hospital based palliative care teams will be trained before the start of the study. This training consists of information on dry mouth characteristics, causes, consequences and treatment options. They will also learn to execute a structured MEP consultation, which includes anamnesis, oral inspection, patient education and treatment advice (Fig. 2). With each participant, these trained nurses will then execute a full MEP consultation on location at the baseline visit, and will monitor the symptoms in a shorter, structured consultation telephonically (or on location) at four different time points in week 2, week 4, week 8 and week 12 (see Fig. 1a).

**Table 1:** Components and content of the Mouth Educational Program (MEP)

Components	Content of the components
**MEP Training**	Interactive training on dry mouth and other oral symptoms, oral care, dry mouth interventions and patient education methods. For all topics involved, see components of the MEP handbook.
**MEP Handbook**	Guidebook to use during the standardized MEP consultations, including road maps, theoretical information and example scenarios
Road maps for standardized consultations	Practical step-by-step plans for both the initial and follow-up consultations, including: 1 Anamnesis 2 Oral examination 3 Patient education 4 Treatment advice 5 Reporting
Theoretical information	Evaluation of dry mouth and other (oral) symptoms • Anamnesis • Oral examination Patient education on dry mouth • Characteristics of dry mouth • Consequences for daily life • Causes of dry mouth Oral care & other interventions • Oral care for patients with varying levels of independence, functionality and frailty • Lifestyle interventions • Causal interventions • Symptom-based interventions • Referrals Monitoring & reporting
Conversation pointers & example scenarios	Practical tips for patient education and shared decision making, and example clinical scenarios with suggestions for treatment plans and patient education topics
**Patient information brochures**	Two brochures with information on oral care and dry mouth respectively

The control condition is CAU, meaning dry mouth care as provided by the regular care team and uninfluenced by the research. No restrictions are placed on the care received and any research procedures, such as administration of questionnaires are handled by our research team.

Usual care practices, within and outside of research settings, are generally characterized by clinical heterogeneity due to differences on the individual professional level and the institutional level in awareness, knowledge, use of guidelines, training, resources, policies and so on [27, 28]. To allow for comparison between a structured form of dry mouth care (MEP) and existing practices (CAU) in two different care settings, for explanation of results, and for identification of barriers and facilitators to implementation, we have decided to collect usual care data through a self-developed questionnaire [27–30].

The institutions allocated to the control group will be offered the MEP training after they have completed the full study period (i.e. included all patients).

#### Pilocarpine study

The pilocarpine study will investigate the effect of locally administered oral pilocarpine drops (3 × 5 mg per day) compared to placebo in reducing complaints of dry mouth in patients with a life-limiting condition or frailty.

Pilocarpine activates the muscarinic receptors, stimulating saliva secretion which in turn alleviates dry mouth symptoms. Systemic pilocarpine tablets have various parasympathomimetic adverse effects such as transpiration, flushing, headaches, dizziness and more [20]. The locally administered oral pilocarpine drops created for this study, however, are topical (non-systemic) and have previously been shown to have minimal adverse effects [21]. In our pilot study an optimal dosage regime was established of 5 mg pilocarpine in drops three times per day [21]. The placebo for this trial has the same qualitative composition, except for pilocarpine HCl, and has comparable traits in terms of texture, taste and look.

For four weeks each group will be instructed to administer 6 drops 3 times a day of either pilocarpine HCL (25 mg/ml) or placebo. Drops are administered left and right outside the molars and in front of the front teeth of the lower jaw, with 2 drops at each site of application. After the blinded 4-week active study period, every participant is offered an 8-week unblinded extension treatment period with off-label pilocarpine eye drops in order to establish longer term efficacy of pilocarpine and to offer active treatment to the placebo group (see also Fig. 1b).

### Study procedures

Both studies in the DROP share the same study time frame (May 2023 – December 2024), time points for data collection and study instruments. Each participant is followed for a 12-week study period with 5 research visits (on *t* = 0, *t* = 2, *t* = 4, *t* = 8 and *t* = 12 weeks) at which questionnaires will be administered (see Table 2 and Fig. 1). At the end of the 12-week study period, a selection of patients will be asked to participate in semi-structured interviews to evaluate the received care. The screening and allocation procedures are different in both studies and are described separately below.

**Table 2:** Data collection schedule and measurement instruments

Data collection	Measurement instrument	Visit 1 *Week 0*	Visit 2 *Week 2*	Visit 3 *Week 4*	Visit 4 *Week 8*	Visit 5 *Week 12*
Baseline characteristics	Questionnaire on patient characteristics	X				
Dry mouth severity	Numeric Rating Scale (NRS) (11-point; from 0 to 10) [1, 31]	X	X	X	X	X
Dry mouth impact	Summated Xerostomia Inventory, shortened Dutch version (sXI-D, adapted) [32]	X	X	X	X	X
Oral health-related quality of life	Geriatric Oral Health Assessment Index, Dutch version (GOHAI-NL) [33]	X	X	X	X	X
Health-related quality of life	EuroQol 5 Dimensions 5 Levels (EQ-5D-5L) [34]	X		X	X	X
Functional status	Patient-reported functional status (PRFS) [35]	X	X	X	X	X
Patient-perceived effect	Global Perceived Effect, 7-point scale [36, 37]		X	X	X	X
Patient-reported medical costs	The Institute for Medial Technology Assessment (iMTA) Medical Consumption questionnaire (iMCQ, adapted) [38]	X		X	X	X
Medication changes	Changes in medication since the last visit		X	X	X	X
Adherence^a^	Short questionnaire on adherence		X	X	X	X
Side-effects^a^	Short questionnaire on side-effects		X	X	X	X

^a^This data is collected in the pilocarpine study only

#### MEP study

Within the MEP study, participating organizations are recruited through a regional network for nursing homes (University Network Care sector Zuid-Holland, UNC-ZH) and interprofessional connections with hospitals and nursing homes. Before the start of the study, all participating organizations are allocated to either the intervention arm (3 nursing homes, 3 hospitals) or control arm (3 nursing homes, 3 hospitals) on a first come, first serve basis (non-randomized).

Upon allocation to the intervention group, the participating nurses will first receive the MEP training before the inclusion of the first patient.

Eligible patients will be identified by the nurses and nurse specialists participating in the study with the help of the regular care teams on the wards of the hospitals and nursing homes. Identified patients will be provided with both oral and written information and an informed consent form on paper. After sufficient time for consideration (a minimum of 24 h), the patient will be asked to participate. Only after written informed consent, the study can commence.

Within the intervention group, all research questionnaires will be administered by the nurses as they are part of the anamnesis within the MEP consultation. Within the control group the questionnaires will be (telephonically) administered by a researcher.

#### Pilocarpine study

Patients will be recruited in two hospitals (one university hospital, one regional hospital) and nursing homes affiliated with The Living Lab in Ageing & Long-Term Care. Recruitment and identification of eligible patients will be carried out by the hospital’s palliative care consultants and nursing homes’ nurses based on the inclusion and exclusion criteria. When an eligible patient has been identified, the care teams will hand over an information letter and the research team will be notified. After a minimum of 1 day, the research team will perform an informative visit to explain the study procedures and the application of the drops, answer any questions and further assess eligibility. After 7 days of deliberation time, formal written informed consent will be obtained. The inclusion procedure will then be followed by (block) randomization of the patient to either pilocarpine or placebo using ALEA®. The randomization outcome will be blinded for both the patient and the researchers. After randomization, the study will officially commence as visualized by Fig. 1.

### Effect evaluation

An overview of the data collection process, including timing of data collection and instruments, is provided in Table 2. All outcome measures and research instruments have been synchronized between the two intervention studies, except for two: the adherence and side-effects questionnaires are only applicable to the pilocarpine study.

#### Baseline measures

Demographic characteristics of patients that will be collected are gender, age, care setting, primary diagnosis and co-morbidities, medication and treatment, presence of (partial) dental prosthesis and use of alcohol and nicotine. In addition, in the MEP study, the demographic characteristics of professionals that will be collected are function, years of experience, palliative care experience and education, and oral care experience and education.

#### Primary outcomes

The primary outcome is the effectiveness of the intervention in reducing dry mouth complaints in patients with a life-limiting condition or frailty. This is measured by the percentage responders at week 4, for which a clinically relevant response is defined as at least a 2-point reduction on the 11-point numeric rating scale (NRS). The NRS is a frequently used instrument in both clinical practice and research settings to score symptom severity, such as for pain, nausea and sleep [31]. The NRS for dry mouth ranges from 0 = no dry mouth symptoms to 10 = worst possible dry mouth symptoms [1, 7].

#### Secondary outcomes

Secondary outcomes include effect durability, (mouth-related) quality of life, patient-reported functional status, global perceived effect by participants, cost-effectiveness, adherence and side-effects. The specific research instruments per outcome used are detailed in Table 2.

Effect durability is measured by the percentage responders at week 8 and 12, as well as by changes in mean NRS dry mouth scores in the intervention versus the control group at all time-points. Effectiveness as perceived by the patient is measured by a 7-point scale (the Global Perceived Effect, GPE) [37] rating how much the patients’ condition has improved or deteriorated since the start of the study in both the control and intervention group. The global perceived effect is measured using a 7-point scale, rating how much the patients’ condition has improved or deteriorated since the start of the study for both the control and intervention group.

Changes in mouth-related quality of life is examined at all time-points between groups using two different validated questionnaires: the Geriatric Oral Health Assessment Index (GOHAI-NL) [33] and the shortened Summated Xerostomia Inventory (sXI-D) [32]. The GOHAI-NL consists of 12 oral health related items and three categories: physical function, psychosocial function and pain/discomfort. The sXI-D consists of 5 items, addressing dry mouth in general and consequences of dry mouth, such as difficulty eating and swallowing. In addition, functional status and overall quality of life are measured by a validated patient-reported functional status scale (from 0 = normal with no limitations to 4 = bed ridden, rarely out of bed) [37] and the EuroQol 5 Dimensions 5 Levels (EQ-5D-5L) questionnaire [34] respectively. The latter will also be used for the cost-effectiveness analysis and assesses quality of life in five dimensions: mobility, self-care, usual activities, pain/discomfort and anxiety/depression. The EQ-5D-5L is complemented by the iMTA (Institute for Medical Technology Assessment) Medical Consumption Questionnaire (iMCQ) for the cost-effectiveness analysis [38]. The iMCQ measures medical consumption, including medical and paramedical treatments or services, in- and outpatient care and medication.

Lastly, in the pilocarpine study the adherence and side-effects from either the pilocarpine or the placebo are measured by using short-form questionnaires.

### Feasibility and acceptability evaluation

The DROP contains a nested qualitative sub-study to evaluate the feasibility and acceptability of either intervention. The main goal is to gain a multi-perspective insight into experiences with current guidelines and care for dry mouth symptoms, into barriers and facilitators within current care and within either intervention, and into feasibility of either intervention. To this end, in both studies, patients will be interviewed about their experiences with the intervention (MEP respectively pilocarpine) or control (CAU respectively placebo) through semi-structured interviews. In addition, in the MEP study, questionnaires on usual care (self-developed) and on implementation of health innovations (validated; [39]) will be administered to participating healthcare professionals. This will then be used to inform topic guides for focus groups which are held separately in each care setting, in both hospitals and nursing homes, and in each arm, in both control and intervention groups.

### Sample size

Sample sizes have been calculated based upon our expectation that both interventions, MEP and pilocarpine, lead to a clinically relevant lower dry mouth score, a minimum 2-point decrease on the 11-point numeric rating scale for dry mouth severity, as compared to CAU respectively placebo.

#### MEP study

For this study, we expect the intervention group (patients who follow the MEP) to have at least 25% more responders (patients with clinically relevant less dry mouth at 4 weeks) than in the control group (patients who receive CAU). To test the null hypothesis (expected difference in responders at 4 weeks of 25%) with a power of 0.8, an alpha of 0.05 and an intra cluster coefficient of 0.10, we will need to include 181 patients which is obtained in 12 clusters (six hospitals and six nursing home clusters). Taking into account a 20% dropout rate at 4 weeks due to the frailty of our study population, 228 patients are needed. Each participating center is therefore expected to include 19 patients.

#### Pilocarpine study

The sample size in this study was based upon our previous pilot study in older people with dry mouth [21]. By using the largest observed standard deviation from the pilot data (i.e. 3.8 points), we calculated a sample size of 57 patients per group, or 114 in total. Considering the low drop-out rates from previous studies with pilocarpine and the knowledge that in our study a less invasive administration method of pilocarpine is used, we expect limited loss-to-follow-up. However, to allow for some drop-out due to the frailty of the study population, the final sample size was set at 120 patients, with 60 patients per group.

### Data monitoring and management

All quantitative data will be collected using the online secure data management system Castor EDC. Of eligible non-consenting patients the reason for non-participation will be collected. The qualitative data from focus groups and interviews will be audio-recorded and transcribed verbatim. The transcripts will be pseudonymized. All data, both study and meta data, will be stored in a secured, digital data safe in either the Maastricht University Medical Center for 25 years (data from the pilocarpine study) or the Leiden University Medical Center for 15 years (data from the MEP study).

### Data analysis

#### Analysis of effect evaluation

All analyses will be carried out using statistical software that supports multi-level mixed model analyses, including the latest version of IMB SPSS Statistics. Demographic and baseline disease characteristic data will be summarized for each treatment group by presenting descriptive statistics. For continuous variables, we will use the mean and standard deviation (SD) in case of a normal distribution, or median and first and third quartile in case of skewed distribution. In case of missing data, we will use stochastic regression imputation to produce a synthetic part of the data to allow for an intention to treat analysis that includes all patients. For all analyses on primary and secondary outcomes a *p* value < 0.05 will be considered to indicate statistical significance. However, interpretation of these significant results will primarily focus on clinical relevance.

The primary outcome (percentage responders at 4 weeks) will be computed by using multi-level logistic regression analysis in the MEP study due to its cluster design, and linear regression analysis in the pilocarpine study taking the randomization stratification variable (sex) into account. Results will be expressed as odds ratio (OR) with a 95% confidence interval (CI).

All secondary outcome measures will be described in full using either mean and standard deviation median and interquartile range for continuous measures or count and proportion for categorical measures. Between-group differences in secondary outcomes at 4, 8, and 12 weeks (i.e., mean GOHAI-NL scores [33], mean PRFS scores [35], and patients’ Global Perceived Effect [37] dichotomized into success and no success) will be tested using multivariable generalized linear regression analysis with a link-function depending on the distribution of the variable (i.e. gaussian, or linear regression for continuous variables, binomial, or logistic regression for binary outcomes).

Cost-effectiveness analysis will be performed to examine both the costs and health outcomes of the interventions versus the control group, using the EQ-5D-5L [34], iMCQ [38] and factual costs of the medicine.

#### Analysis of feasibility and acceptability

All qualitative data from the nested mixed-methods sub-study on the feasibility and acceptability of the MEP in comparison to CAU, i.e. transcriptions and field notes of the focus groups and interviews, will be analyzed through thematic analysis [40]. Thematic analysis will be performed by two researchers independently using qualitative data analysis software, such as Atlas Ti, and will be further discussed in consensus meetings.

### Ethical considerations

Both studies within the DROP have been reviewed by independent medical ethical boards and have been found to have a limited risk and burden profile.

#### MEP study

The MEP study was reviewed by the Medical Ethics Committee of the Leiden University Medical Center and was approved as a non-WMO research project. No risks are to be expected by participating as the education program solely consists of information and treatment advice from existing guidelines and standard care practices in the Netherlands. Burden may be found in the effort it may take to answer questionnaires in combination with a frail study population (i.e. older and/or patients with life limiting illnesses). However, this level of burden has been minimized by limiting the frequency of, the length of and the sensitive topics within the questionnaires.

#### Pilocarpine study

The pilocarpine study was submitted through the new Clinical Trials Information System (CTIS) and approved by the Medical Ethics Committee of Maastricht University Medical Center + . The risk classification analysis showed that the pilocarpine study entails moderate risk since pilocarpine is a registered drug for oral and ocular use. Case-reports and case-series indicate that topical pilocarpine administration has none or minor side effects (irritation of mouth and tongue). The burden of xerostomia on patient well-being outweighs the risk of side effects. Small studies and case reports suggest that local application has none or only minor side effects [20]. In the experimental group, benefits of participation may include relief of dry mouth symptoms and improved (oral-health-related) quality of life. Similar to the experimental group, the control group, will be offered unblinded expanded access to pilocarpine eye drops after 4 weeks and may therefore benefit from similar results.

## Discussion

This study protocol describes the DROP, a comprehensive research project investigating a non-pharmacological intervention, the Mouth Education Program (MEP), and a pharmacological intervention, pilocarpine, for dry mouth in patients with a life-limiting illness or frailty. Both interventions are examined in large-scale, multi-center trials that focus on the short-term and long-term effects in reducing dry mouth symptoms, influence on quality of life, cost-effectiveness and feasibility.

The non-pharmacological, nurse-led patient education program (the MEP) was co-created with researchers and health care professionals of different backgrounds, thus ensuring a strong scientific, clinical and practical foundation. In addition, we built upon previous experience with nurse-led education programs within [26] and outside our research group [15, 16]. As palliative care is an integral part of all health care, not just hospital-based or just nursing home-based care, the intervention is tested in both care settings (hospital, nursing home) and within different nurse fields (nurse specialists, nurses and nurse assistants). A cluster design was chosen to prevent between-condition contamination and simplify participation for health care professionals and their organizations.

While using a different study design, similar methodological, theoretical and practical considerations were made in the pharmacological study. Using a double-blind placebo-controlled randomized trial to investigate the effectiveness of topical pilocarpine administration ensures a high evidence level while simultaneously minimizing response and selection bias. Furthermore, participants in the pilocarpine study will also be recruited from both nursing home and hospital settings to increase generalizability of findings.

Moreover, the qualitative sub-study in both the MEP and pilocarpine study, evaluating the feasibility and usability of the interventions by both the patients’ and health care professionals’ perspectives, contributes to a strong foundation for future implementation in clinical practice. It will provide us with valuable information on the interventions’ facilitators and barriers versus those of the comparators (care as usual and placebo). To stimulate the wide-spread implementation and uptake of the interventions, an intensive dissemination plan has been developed which includes, but is not limited to, publication in scientific journals, active outreach to national palliative care stakeholders in the form of meetings and presentations and integration into the Dutch palliative care guideline for oral symptoms [18]. In addition, the MEP will also be made freely accessible after completion of the study.

### Limitations

This study does come with some methodological challenges. Recruitment difficulty and loss-to-follow-up due to frailty, deteriorating condition or death are specifically of concern with this palliative study population. By excluding terminally ill patients (life expectancy of 4 weeks or shorter), limiting the number of questionnaires (thus participation burden) and increasing the sample sizes, we hope to limit the impact of this concern.

However, recruitment is not only a concern within the patient population. The post-covid health care system is under great pressure in the Netherlands. Increased healthcare consumption paired with staffing problems due to high burn-out and other illness absence rates have led to increased work pressure and vice versa. This may impact health care professionals’ willingness to participate in the MEP study for which active participation and an additional time investment is needed. We attempt to address this pre-emptively by offering intensive and easily accessible support as well as a monetary stipend per included patient. As for the pilocarpine study, patients are provided with one-on-one instructions by the researchers to administer the drops themselves, to relieve their regular care team of potential extra tasks.

Regarding the cluster non-randomized design of the MEP study, a balance was sought between methodological strength and logistic constraint. A non-randomized design enables us to include care institutions more swiftly and easily (on a rolling basis) within a limited time frame, but may bring in some level of response bias. However, its cluster design limits the level of bias as it merely takes place on an organizational level, not on the patient or clinician level as with a patient-randomized trial.

## Conclusion

Dry mouth is a prevalent and impactful yet little acknowledged symptom in the last phase of life. Hence, the DROP intends to increase awareness and knowledge in both patients and health care professionals and to contribute to more effective treatment options. This research project will investigate two new interventions, a nurse-led patient education program and the topical administration of pilocarpine, and will examine the effectiveness, change in quality of life and feasibility of either intervention. The DROP has started recruitment in June 2023 and is expected to be completed at the end of 2024.

## Data Availability

Not applicable.

## Abbreviations

CAU: Care as Usual
MEP: Mouth Education Program
